# RETRACTED: Kozlov et al. Perfluorinated Sulfonic Acid-Based Ionomers: Current State and Prospects. *Polymers* 2026, *18*, 848

**DOI:** 10.3390/polym18192384

**Published:** 2026-09-30

**Authors:** Valeriy A. Kozlov, Barry W. Ninham, Sergey M. Kuznetsov, Sergey V. Gudkov, Nikolai F. Bunkin

**Affiliations:** 1 Prokhorov General Physics Institute of the Russian Academy of Sciences, Vavilov Str. 38, 119991 Moscow, Russia; v.kozlov@hotmail.com (V.A.K.); kuznetsov.sm.93@gmail.com (S.M.K.); s_makariy@rambler.ru (S.V.G.); 2 Department of Fundamental Sciences, Bauman Moscow State Technical University, 5 2nd Baumanskaya St., 105005 Moscow, Russia; 3 Materials Physics (Formerly Department of Applied Mathematics), Research School of Physics, Australian National University, Canberra, ACT 2600, Australia; barry.ninham@anu.edu.au

The journal retracts the article titled “Perfluorinated sulfonic acid-based ionomers: current state and prospects” [1], cited above.

Following publication, concerns were brought to the publisher’s attention regarding an overlap between this paper [1] and an earlier publication produced by a different authorship group [2].

Adhering to our standard procedure, an investigation was conducted by the Editorial Office and Editorial Board. While the authors fully cooperated with the investigation through the clarification of the raised concerns, the Editorial Board confirmed a significant overlap between these publications [1,2], without the appropriate acknowledgement and citation. Given the scale of the overlap, the Editorial Office and the Editorial Board have decided to retract this article as per MDPI’s retraction policy.

This retraction was approved by the Editor-in-Chief of the journal *Polymers*.

The authors have been informed of this retraction and have indicated their disagreement with this decision.
